# Transcriptomic Analysis of Host Immune and Cell Death Responses Associated with the Influenza A Virus PB1-F2 Protein

**DOI:** 10.1371/journal.ppat.1002202

**Published:** 2011-08-25

**Authors:** Ronan Le Goffic, Olivier Leymarie, Christophe Chevalier, Emmanuelle Rebours, Bruno Da Costa, Jasmina Vidic, Delphyne Descamps, Jean-Michel Sallenave, Michel Rauch, Michel Samson, Bernard Delmas

**Affiliations:** 1 Unité de Virologie et Immunologie Moléculaires, UR 892 INRA, Jouy-en-Josas, France; 2 Plateforme d'instrumentation et de Compétences en Transcriptomique, Jouy-en-Josas, France; 3 Unité de Défense Innée et Inflammation, Institut Pasteur, Paris, France; 4 Unité U874 INSERM, Paris, France; 5 Université Paris 7-Denis Diderot, Paris, France; 6 EA 4427 SeRAIC, Université de Rennes 1, Rennes Cedex, France; St. Jude Children's Research Hospital, United States of America

## Abstract

Airway inflammation plays a major role in the pathogenesis of influenza viruses and can lead to a fatal outcome. One of the challenging objectives in the field of influenza research is the identification of the molecular bases associated to the immunopathological disorders developed during infection. While its precise function in the virus cycle is still unclear, the viral protein PB1-F2 is proposed to exert a deleterious activity within the infected host. Using an engineered recombinant virus unable to express PB1-F2 and its wild-type homolog, we analyzed and compared the pathogenicity and host response developed by the two viruses in a mouse model. We confirmed that the deletion of PB1-F2 renders the virus less virulent. The global transcriptomic analyses of the infected lungs revealed a potent impact of PB1-F2 on the response developed by the host. Thus, after two days post-infection, PB1-F2 invalidation severely decreased the number of genes activated by the host. PB1-F2 expression induced an increase in the number and level of expression of activated genes linked to cell death, inflammatory response and neutrophil chemotaxis. When generating interactive gene networks specific to PB1-F2, we identified IFN-γ as a central regulator of PB1-F2-regulated genes. The enhanced cell death of airway-recruited leukocytes was evidenced using an apoptosis assay, confirming the pro-apoptotic properties of PB1-F2. Using a NF-kB luciferase adenoviral vector, we were able to quantify *in vivo* the implication of NF-kB in the inflammation mediated by the influenza virus infection; we found that PB1-F2 expression intensifies the NF-kB activity. Finally, we quantified the neutrophil recruitment within the airways, and showed that this type of leukocyte is more abundant during the infection of the wild-type virus. Collectively, these data demonstrate that PB1-F2 strongly influences the early host response during IAV infection and provides new insights into the mechanisms by which PB1-F2 mediates virulence.

## Author Summary

Influenza A viruses may cause severe respiratory disease. PB1-F2, a viral protein identified in 2001 is suspected to play a role in influenza-related pneumonia. In order to understand the impact of PB1-F2 in the pathogenesis underlying Influenza A virus infection, we engineered a mutant virus unable to express PB1-F2. By the use of high-throughput gene expression assays, we compared the host responses of the wild-type-infected and the PB1-F2 mutant-infected mice. We identified that PB1-F2 expression enhances the immune cell death and inflammatory responses of mice. The inflammatory response mediated by the PB1-F2 expression leads to a massive recruitment of leukocytes within the air spaces, a feature that characterizes the influenza-mediated immunopathology. Our results suggest that PB1-F2 is a virulence factor implicated in the deregulation of the inflammatory response observed in acute influenza virus pneumonia. These data underlie the complexities of virus-host interactions and help us understand by which mechanisms Influenza viruses mediate severe respiratory diseases.

## Introduction

Influenza A virus (IAV) commonly causes acute respiratory infection and is one of the most important human pathogens, causing between 250,000 and 500,000 deaths every year around the world [1]. IAV are enveloped viruses belonging to the *Orthomyxoviridae* family. Their negative strand RNA genome is composed of 8 segments encoding up to 12 proteins. PB1-F2 is a virulence factor first described 10 years ago [2]. This 75–90 amino acid long accessory protein is encoded by an alternative +1 reading frame on segment 2 which also encodes the RNA polymerase basic protein 1 (PB1) and N40, an N-terminally truncated version of PB1 lacking transcriptase function [2], [3]. PB1-F2 is expressed by most IAV strains and has been shown to be associated with immunopathological processes observed during infection [4], [5], [6]. During IAV infection, production of pro-inflammatory cytokines generally results in an innate host response that controls the virus propagation until it is eliminated by the adaptive immune system. However, in some cases, excessive host inflammatory response contributes to disease severity, especially with highly pathogenic strains such as avian H5N1 or the 1918 pandemic H1N1 strain (“Spanish flu”) [7], [8]. PB1-F2 is suspected to contribute to this disproportional response which frequently leads to vital respiratory tissue damage and death of the infected person [5].

Several *in vivo* studies allowed determining the pathophysiological consequences of PB1-F2 expression during IAV infection. Using an attenuated model of mouse infection, Zamarin *et al.* demonstrated that wild type (wt) IAV displays a higher pathogenicity than its PB1-F2 knockout counterpart [9]. A single N66S mutation present in the PB1-F2 of the 1918 pandemic strain is sufficient to transform a strain of moderate virulence into a highly pathogenic virus in mice [10]. More recently, a study analyzing the effects of PB1-F2 in the IAV-induced pathogenesis of avian hosts also revealed that amino acid changes within the PB1-F2 reading frame of a highly pathogenic avian IAV decrease lethality in ducks [11]. PB1-F2 was also described as facilitating secondary infection with *Streptococcus pneumoniae* [12]. In contrast, when introducing a functional PB1-F2 in the 2009 pandemic H1N1 IAV that does not encode this accessory protein, only minimal immune response modulation was observed, underlying the complex contribution of PB1-F2 in virulence [13].

We recently showed that PB1-F2 exacerbates IFN-β production through the activation of the NF-κB pathway during IAV infection of the human respiratory epithelial cell line A549, but not during infection of immune cells [6]. Despite its apoptotic functions, pathways related to cell death were not activated differently in wt and PB1-F2 knockout virus-infected A549 cells. In the present study, we analyzed the global transcriptional response of the mouse respiratory tract associated to PB1-F2 during IAV infection. We report that PB1-F2 expression during IAV infection increases the inflammatory response of the host. PB1-F2 plays a major role in NF-κB pathway activation, chemotaxis of granulocyte and apoptosis of recruited immune cells. Furthermore, we determined that IFN-γ expression is enhanced when PB1-F2 is expressed, and appears to have a central position in the gene network responsible for the respiratory disease provoked by IAV.

## Results

### Kinetics of PB1-F2 protein expression in infected mice and associated-pathogenicity

**Figure 1:**
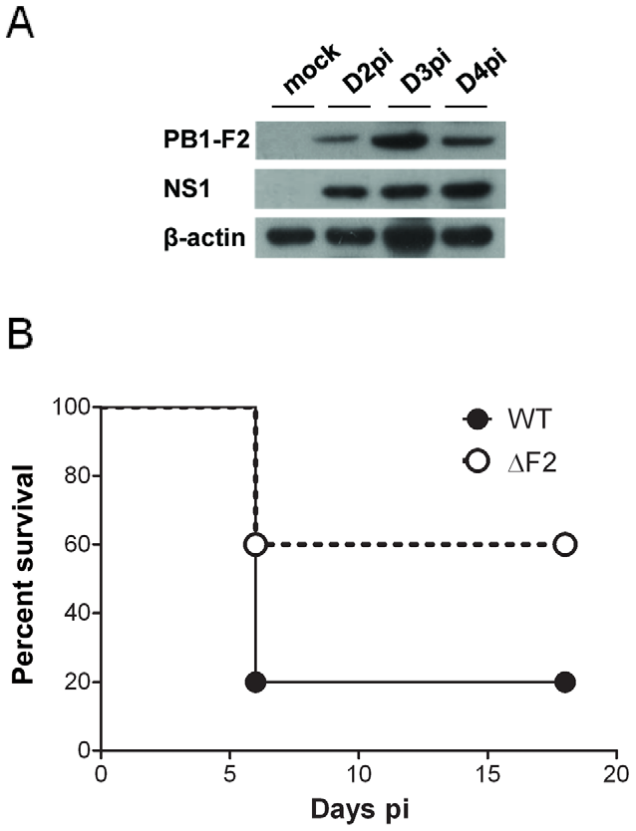
Expression of PB1-F2 and associated pathogenicity in infected mice. (A) In vivo kinetics of PB1-F2 expression in IAV-infected mice lungs. C57Bl/6 mice were infected with 1.5×10^5^ PFU of IAV strain A/WSN/33. Lungs were collected at different time points pi and lysates were subjected to Western blot using antisera against PB1-F2 and NS1. β-actin was used as a loading control. (B) Lethality induced by wt and ΔF2 IAV in C57Bl/6 mice. Mice received intranasally 2.5×10^5^ PFU of wt or ΔF2 IAV and were observed daily for signs of morbidity.

In order to analyze the impact of PB1-F2 expression in pathogenicity, we first examined the kinetics of PB1-F2 expression in a mouse infection model using the mouse-adapted A/WSN/1933 (H1N1) strain of IAV. The time course of PB1-F2 expression in the lungs of infected mice was analyzed. PB1-F2 was efficiently expressed at day 2 post-infection (pi) and expression increased at day 3 and 4 pi (Fig. 1A). Densitometric measurement of PB1-F2 levels standardized to β-actin levels on distinct infected mice confirmed the increase of PB1-F2 expression at days 3 and 4 pi (Fig. S1). Using a sensorchip assay (Biacore) with an anti-PB1-F2 monoclonal antibody [14], the PB1-F2 concentration was estimated to range around 4 pmol per 100 pmol of NP at day 4 pi, corresponding to 26 pmol of PB1-F2 per mg of infected lung tissue.

We then aimed to determine the specific effect of PB1-F2 expression on the mouse mortality rate using the wt virus and a mutant knocked-out for PB1-F2 expression. To this end, and because the introduction of mutations at the initiation and internal AUG codons of the PB1-F2 open reading frame (ORF) may result in the generation of truncated PB1 products [2], [3], we generated mutants unable to reinitiate translation in the two ORFs: PB1-F2-null and N40-null mutants (Fig. S2A). We then compared the effect of PB1-F2- and N40-null mutations on PB1 expression in the infected mouse epithelial cell line MLE15 in order to verify that all viruses expressed similar amounts of PB1 (Fig. S2B). Furthermore, to determine if N40 expression may modify the host immune response, infections of MLE15 cells by the wt virus and its mutants were carried out and IFN-β synthesis induction analyzed. As expected, the N40 null-mutants induced similar amounts of IFN-β than the wt virus, allowing us to exclude the involvement of N40 in host-response exacerbation processes (Fig. S2C). Therefore, we retained the fully knocked-out mutant ΔF2 for further mice infections and subsequent analyses [6].

While 100% mortality was reached when mice were inoculated with 1×10^6^ PFU with the wt and the ΔF2 viruses, the 50% Lethal Doses (LD50) were estimated to be 1.48×10^5^ PFU [95% confidence interval (CI), 0.97×10^5^ - 1.9×10^5^] with the wt virus and 2.81×10^5^ PFU [CI, 2.6×10^5^ - 2.99×10^5^] with the ΔF2 virus. No mortality was observed when mice were inoculated with 5×10^4^ PFU of the two viruses. A survival curve after a viral challenge using 2.5×10^5^ PFU is shown in Fig.1B. A lower mortality rate was observed in the ΔF2 infected mice when compared to the wt infected mice (40% vs. 80%). Immunohisto-labelling of NS1 in mouse-infected lungs showed equivalent signal intensities for the two viruses indicating an identical viral infectivity (Fig. S3). These data are indicative of an impact of PB1-F2 expression in IAV-induced pathogenicity.

### PB1-F2 causes increased morbidity and enhanced IFN-β expression

**Figure 2:**
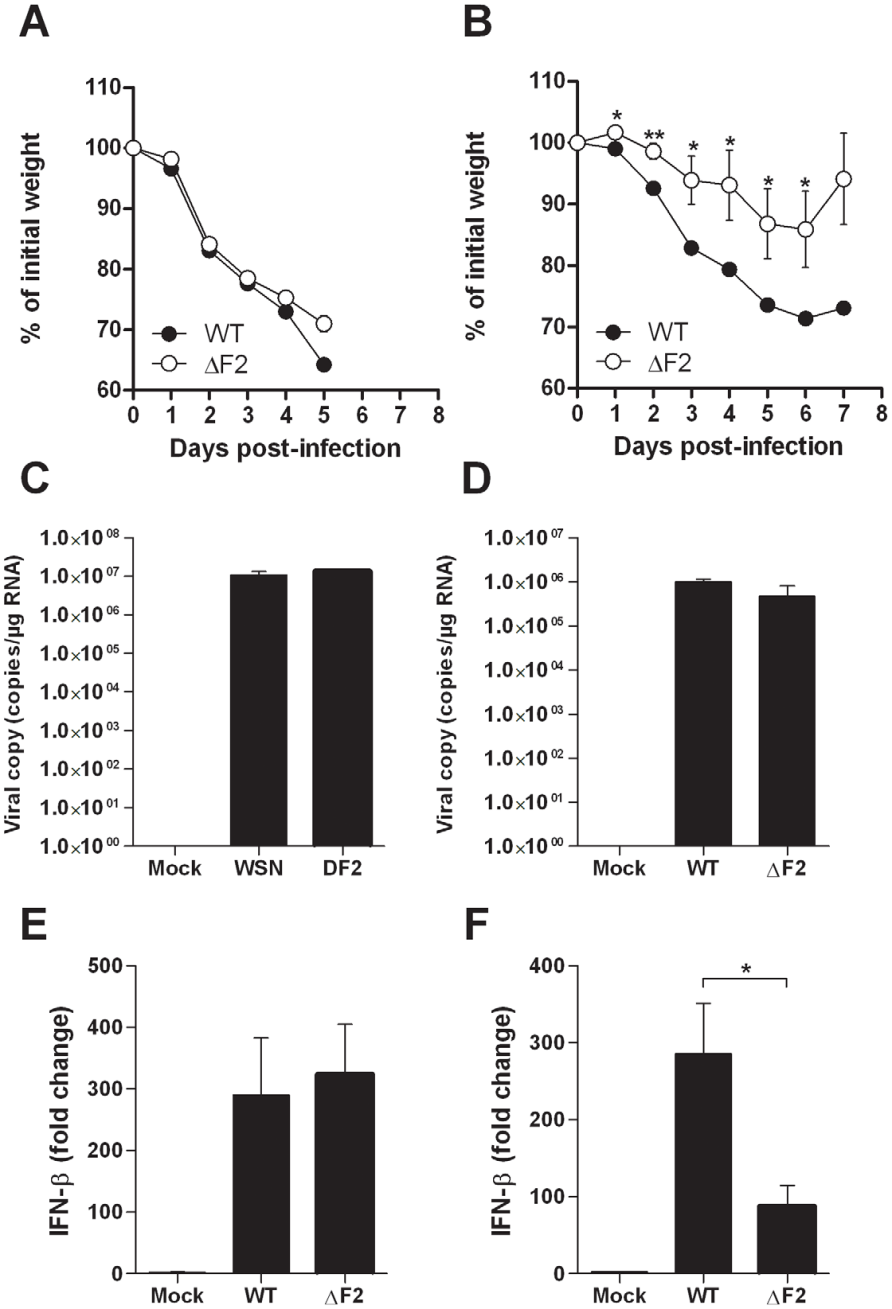
Effect of PB1-F2 expression on pathogenicity during IAV infection. (A-B) Time-course of body weight changes, mice were inoculated with 1×10^6^ PFU (A) and 5×10^4^ PFU (B) and their weights measured every day. (C-D) Viral load in lungs of mice challenged by 1×10^6^ PFU (C) and 5×10^4^ PFU (D) of wt and ΔF2 IAV. Results are the mean ± SD values obtained from 4 animals at day 3 pi. They are expressed as RNA copies normalized to the quantity of RNA used in the experiment. (E–F) Real time RT-PCR quantification of IFN-β gene expression in lungs of mice infected by 1×10^6^ PFU (E) and 5×10^4^ PFU (F) of wt and ΔF2 IAV 3 days pi. IFN-β expression was normalized with the β-actin gene expression level and presented as fold increase relative to mock-treated mice. (* p<0.05; **p<0.01).

To investigate the contribution of PB1-F2 in virus pathogenicity, we infected groups of mice at high and low infectious doses to measure and compare morbidities, replication levels and IFN-β inductions. Measurement of IFN-β was carried out since we previously observed that PB1-F2 exacerbates IFN-β expression within *in vitro* infected human pulmonary cells [6]. In response to a high viral challenge (1×10^6^ PFU), the two groups of mice (wt- and ΔF2-infected mice) were as sensitive (0% survival for both groups) and lost the same weight (Fig. 2A). When infections were carried out with lower sub-lethal doses of virus (5×10^4^ PFU), wt-infected mice lost a greater percentage of body weight than their ΔF2-infected counterparts: 29% vs. 14% at day 6 pi (Fig. 2B). The differential weight loss persisted during the viral infection. To determine whether the morbidity associated to PB1-F2 expression could be attributed to a reduced viral replication in ΔF2 infected mice, we assessed viral RNA in the lungs by qRT-PCR. As shown in Fig. 2C and 2D, at day 3 pi, both viruses replicate with the same efficiencies when mice were infected with a high or a low viral dose. Wt- and ΔF2-virus titers were also identical at days 2 and 4 pi (data not shown). These observations are consistent with previous studies [6], [9]. We then measured the level of induction of IFN-β gene transcription at day 3 pi in the two conditions of viral challenge. When mice were infected with 1×10^6^ PFU, both viral infections induced a strong IFN-β expression with the same range of magnitude (Fig. 2E). At the lower infection dose, while IFN-β gene transcription is still strongly induced in wt-infected mice, a reduced fold of induction was observed in ΔF2-infected mice (Fig. 2F). Collectively our results indicate that, as we previously observed in cultured human epithelial pulmonary cells, PB1-F2 is able to exacerbate IFN-β expression in infected mice.

### Comparison of the mouse lung response to wt and ΔF2 IAV mutant

**Figure 3:**
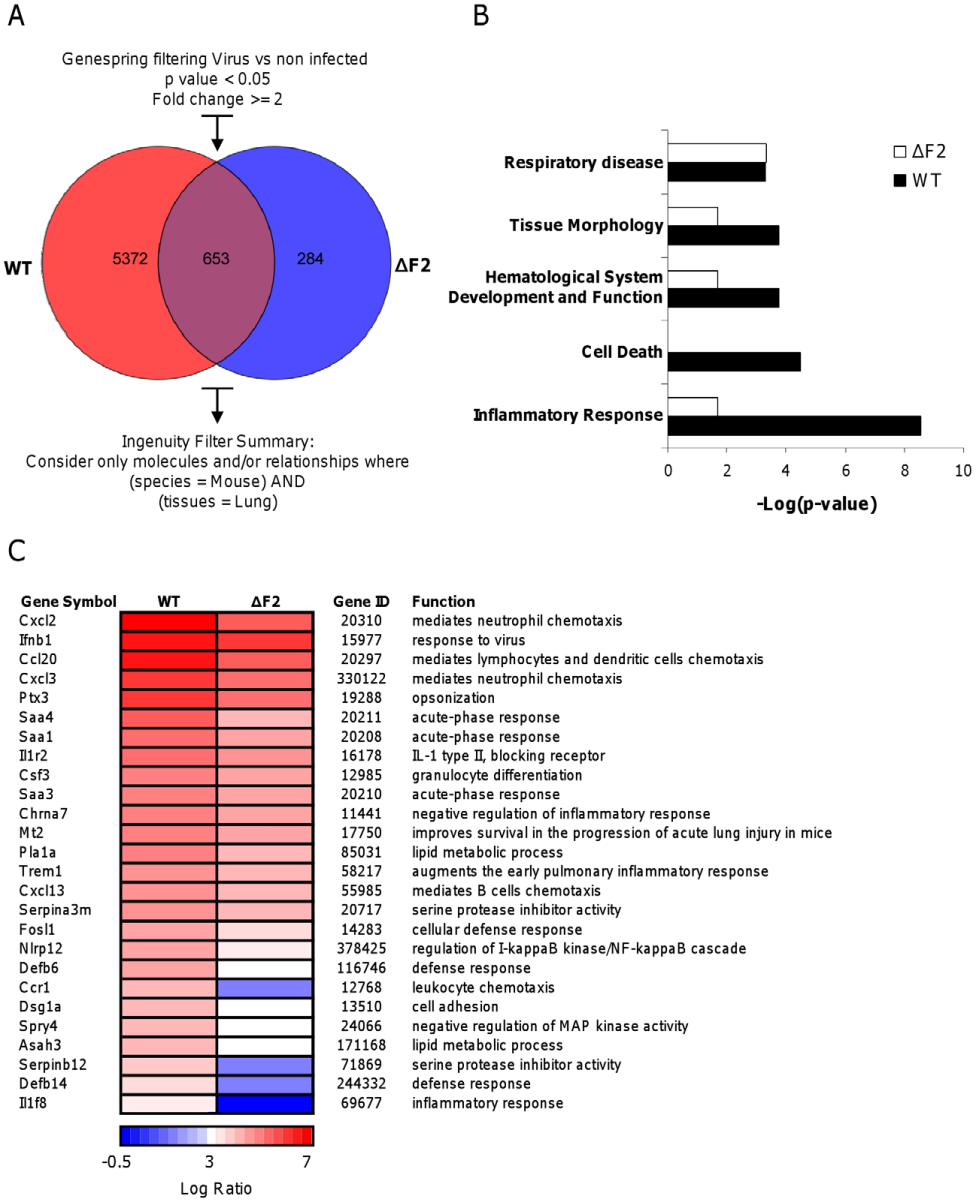
Microarray analysis of gene expression in the lungs of mice on day 2 pi. (A) Venn diagram showing the distribution of differentially regulated genes during infection with wt or ΔF2 IAV relative to mock-infected mice. (B) Functional categories comparison of differentially expressed genes in lungs of mice infected with wt or ΔF2 IAV. (C) Heat map of selected genes related to innate defense in the lungs of wt- and ΔF2-infected mice at day 2 pi based on analysis using Genespring software (Agilent). Genes shown in red are up-regulated and those shown in blue are down-regulated in infected mice compared to mock-infected mice. Data are expressed in Log base 2 ratio.

To provide additional insights on the pathogenicity associated to PB1-F2 expression during IAV infection, we investigated transcriptional profiles of the host genes in the lungs of mice infected by the wt- and the ΔF2-virus by microarray analyses. Mice were infected intranasally with 1.5×10^5^ PFU and total RNA were extracted from mice lungs at day 2, 3, and 4 pi and analyzed by microarrays containing more than 44,000 oligonucleotides representative of the whole murine transcriptome. The transcriptomic profiles obtained at day 2 pi showed the most relevant differences between the two infection conditions. A Venn diagram shown in Fig. 3A summarizes the differentially expressed genes common or unique to wt and ΔF2 virus infections at day 2 pi. A total of 6025 genes showed a greater-than-2-fold up- or down-regulation in wt-infected lungs relative to mock-infected lung (p<0.05). By contrast, when looking at the regulated-genes during the infection with the ΔF2 virus, only 937 genes were regulated, demonstrating that PB1-F2 severely influence the early host response during IAV infection of mice. The most significant canonical pathways specifically associated with wt and ΔF2 virus infections according to Ingenuity Pathway Analysis (IPA) software are listed in Fig. 3B. The analysis revealed that both viruses induced genes involved in “Respiratory disease” showing that wt and ΔF2 viruses are both able to establish a deleterious infection in C57Bl/6 mice. Genes involved in the “Cell death” pathway are expressed at high levels in mice infected by the wt virus while it is not statistically regulated in ΔF2-infected mice. This is consistent with the fact that PB1-F2 is described as a proapoptotic factor in immune cells [2]. Another main functional consequence of the PB1-F2 deletion observed is the drastically reduced inflammatory response developed during the ΔF2 virus infection. This suggests a potent pro-inflammatory activity of PB1-F2 that could be an important contributor to severe immunopathology associated with highly pathogenic IAV strains. We also observed a correlation between PB1-F2 expression and the pathways “Tissue Morphology” and “Hematological System Development and Function”, which are direct consequences of the inflammatory response. As shown in Fig.3C, genes implicated in inflammatory processes were shown to be preferentially regulated during the wt IAV infection, including genes involved in chemotaxis and activation of granulocytes (Cxcl2, Cxcl3, Csf3, Trem1), genes encoding acute-phase proteins (Saa1, Saa3 and Saa4) and multiple other genes implicated in defense response. In order to validate the microarray expression data, we assayed several genes presented in Fig. 3C by qRT-PCR (Ccr1, Cxcl1, Csf3, Ptx3, Tnf-α and Trem1). These experiments performed on independent mice confirmed the PB1-F2 effect on inflammatory genes (Fig. S4). Collectively, these results suggest that PB1-F2 amplifies the inflammation observed during IAV infection, a feature that was partially identified in our previous analysis using the A549 cell infection model.

### PB1-F2 expression induces an increase in inflammatory mediator secretions during IAV infection

**Figure 4:**
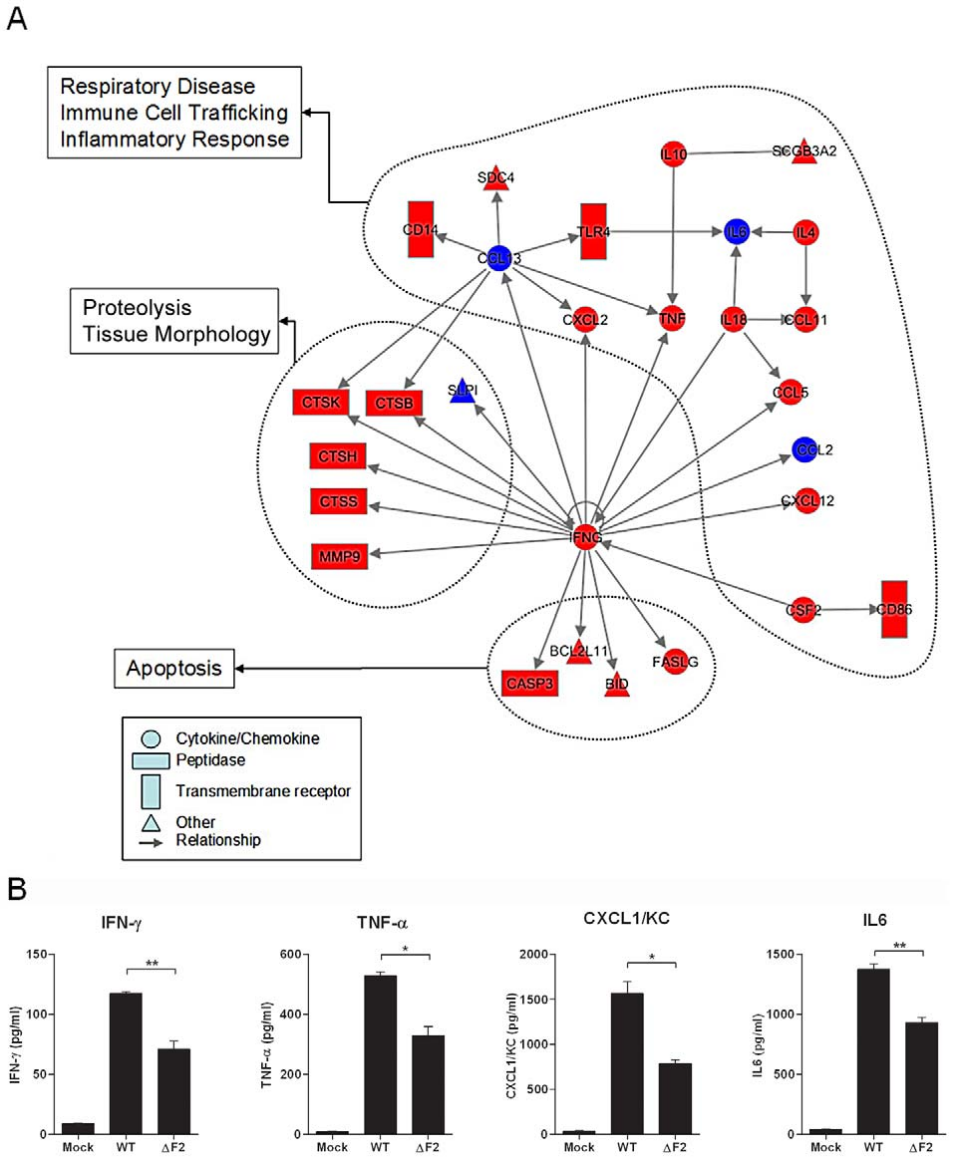
Gene network analysis. (A) Biological network of genes that were specifically regulated more than 2 fold (P<0.05) in the wt-infected mice lungs compared with mock-infected controls. Lines between nodes in the network represent gene interactions. Subset of genes that were regulated during wt IAV infection but not during ΔF2 IAV infection are shown in red, and genes expressed at a high level in the two infectious conditions are shown in blue. Biological networks were made by using Ingenuity Pathway Analysis. The meaning of the symbols is indicated in the inset of the figure. (B) BAL fluids levels of IFN-γ, TNF-α, CXCL1/KC and IL-6 in mock-infected, wt-infected and ΔF2-infected mice at day 2 (IFN-γ) or day 3 (TNF-α, CXCL1/KC and IL-6) pi (*p<0.05, **p<0.01).

To further explore the host responses associated with PB1-F2 expression, we characterized the functional relationship between genes that were induced or inhibited more than two fold (p<0.05) between wt- and ΔF2-infected mice. Fig. 4A shows a representation of the significant gene network associated with PB1-F2 expression during mice infection. Top functions associated with this gene network were related to immune cell trafficking, inflammatory response and cell death. Of particular interest is the central position of IFN-γ in this network. IFN-γ is linked to multiple genes including peptidases, apoptotic activators, chemokines and cytokines. Most of these genes are involved in immune cell functions or in recruitment of monocytes and granulocytes towards sites of tissue infection.

To confirm the ability of PB1-F2 to promote an increase in inflammatory mediator secretion during infection, we dosed by ELISA the amount of several cytokines involved in the inflammation process in the bronchoalveolar lavage (BAL) fluid of infected animals. Thus, IFNγ was quantified due to its central position within the network, TNFα because of its potent role in inflammatory processes, CXCL1/KC for its important role in neutrophil chemoattraction and IL6 as the most important mediator of fever and for its role in the acute phase response. As shown in Fig. 4B, these four cytokines were present in lower amounts in the BAL of mice infected by the ΔF2 virus when compared with BAL obtained from animals infected with the wt virus. These statistically relevant observations further confirmed the pro-inflammatory capacities of PB1-F2 during IAV infection.

Altogether, our results indicate that upon influenza infection, the respiratory tract exhibits increased lysosomal proteases, leucocyte chemoattractants, and inflammatory signaling mRNA transcripts when PB1-F2 is expressed and suggest that this viral protein plays a major role in acute lung inflammatory processes and might be involved in severe immunopathology.

### Apoptosis of BAL immune cells is mediated by PB1-F2

**Figure 5:**
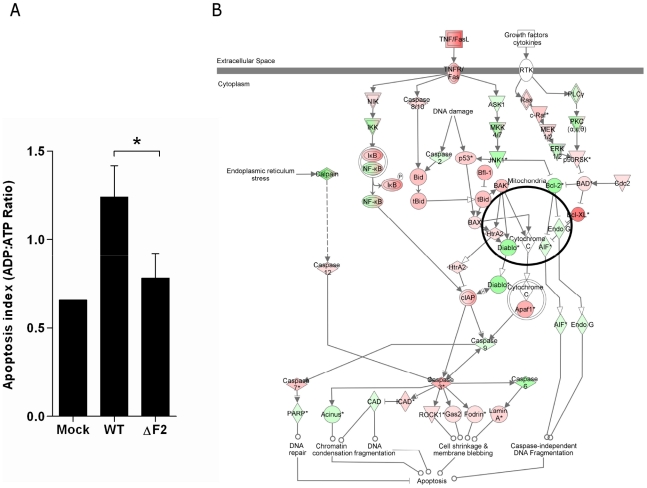
Apoptotic index of leukocytes present in the BAL fluids of IAV-infected mice. (A) Cells from BAL fluids of IAV-infected mice were harvested after 3 days pi. Apoptotic index was assayed by measuring the ADP:ATP ratio (*p<0.05). (B) Ingenuity Systems software was used to represent regulation of genes involved in apoptotic pathway during wt IAV infection of mice lungs. Genes depicted in red are increased and genes in green are decreased compared with mock-infected mice. Color intensity increases with the magnitude of fold-change.

We sought to verify the microarray results by assessing apoptosis in cells found in BAL fluids. BAL total cells were harvested at day 3 pi and the apoptotic index monitored. We opted for a method allowing the distinction between necrosis and apoptosis, and giving a result in the form of a ratio, excluding differences in terms of number of cells. As shown in Fig. 5A, the apoptotic index of cells recruited in the airways of wt-infected mice is significantly increased when compared to the mock-infected mice. Conversely, when apoptosis quantification was made in ΔF2-infected mice, we found that the apoptosis index was significantly reduced when compared to wt-infected mice. We next used the IPA software to identify the apoptosis-related signalling pathways activated during wt IAV infection of mice lungs. As shown in Fig.5B, multiple genes involved in the pathway linked to TNF/FasL-mediated apoptosis are induced (red) when mice were infected with the wt virus, including numerous caspases. Apoptotic genes induced under these conditions suggest that the PB1-F2-mediated apoptosis response of the infected lung is mediated by activation of genes promoting cell shrinkage and membrane blebbing.

### PB1-F2 expression mediates an exacerbation of NF-κB activation during infection

**Figure 6:**
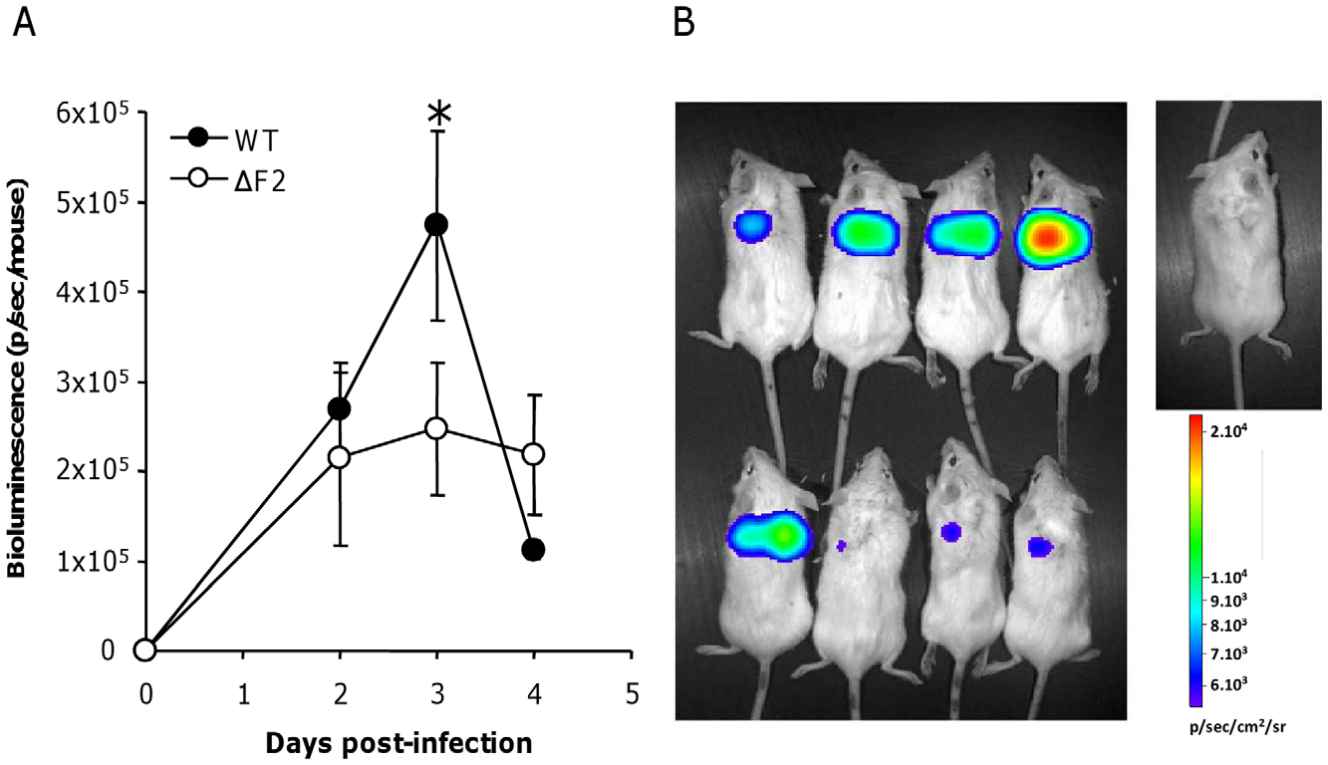
NF-κB luminescence in the lungs of wt and ΔF2 IAV-infected mice. (A) Groups of 4 Balb/c mice were co-infected at day 0 by either wt or ΔF2 IAV (1.5×10^5^ PFU per mouse) and by a replication-deficient adenovirus used to transduce NF-κB-luciferase construct (Ad-NF-κB-luc). Bioluminescence was then measured daily by intranasal injection of 50 µl of luciferine (500 µg/ml) and capture of photon emission from the chest at different times post-infection using the IVIS system. (B) Pictures of anesthetized mice after luciferine instillation taken 3 days pi. The scale on the right indicates the average radiance: the sum of the photons per second from each pixel inside the ROI/number of pixels (photons/sec/cm^2^/sr).

NF-κB is the major pro-inflammatory transcriptional factor and regulates the induction of most proinflammatory cytokines. We recently observed that PB1-F2 mediates an upregulation of IFN-β by NF-κB activation in human pulmonary cells [6]. We thus analyzed the contribution of PB1-F2 in the NF-κB activation during an *in vivo* IAV infection. We used an adenovirus containing an NF-κB response element linked to a luciferase reporter gene (Ad-NF-κB-luc) [15]. As a positive control, we showed that E. Coli LPS induces NF-κB activation (Fig. S5). Control mice infected with Ad-NF-κB-luc alone (PBS) showed no NF-κB activity, attesting that the vector has no inflammatory activity by itself. Mice were co-infected with the Ad-NF-κB-luc and either the wt IAV or the ΔF2 IAV (1.5×10^5^ PFU per mouse). Mice were then followed daily for luciferase activity measurement (Fig. 6A). Wt-infected mice developed an important inflammatory reaction with a peak of activity after 3 days pi in contrast to animals infected by the ΔF2 virus that developed a moderate NF-κB activity (Fig. 6B). Quantification of the luciferase activity showed that the difference is observable at day 3 pi, which corresponds to the viral replication peak (data not shown).

### Differential immune cell recruitment between wt- and ΔF2-infected mice

**Figure 7:**
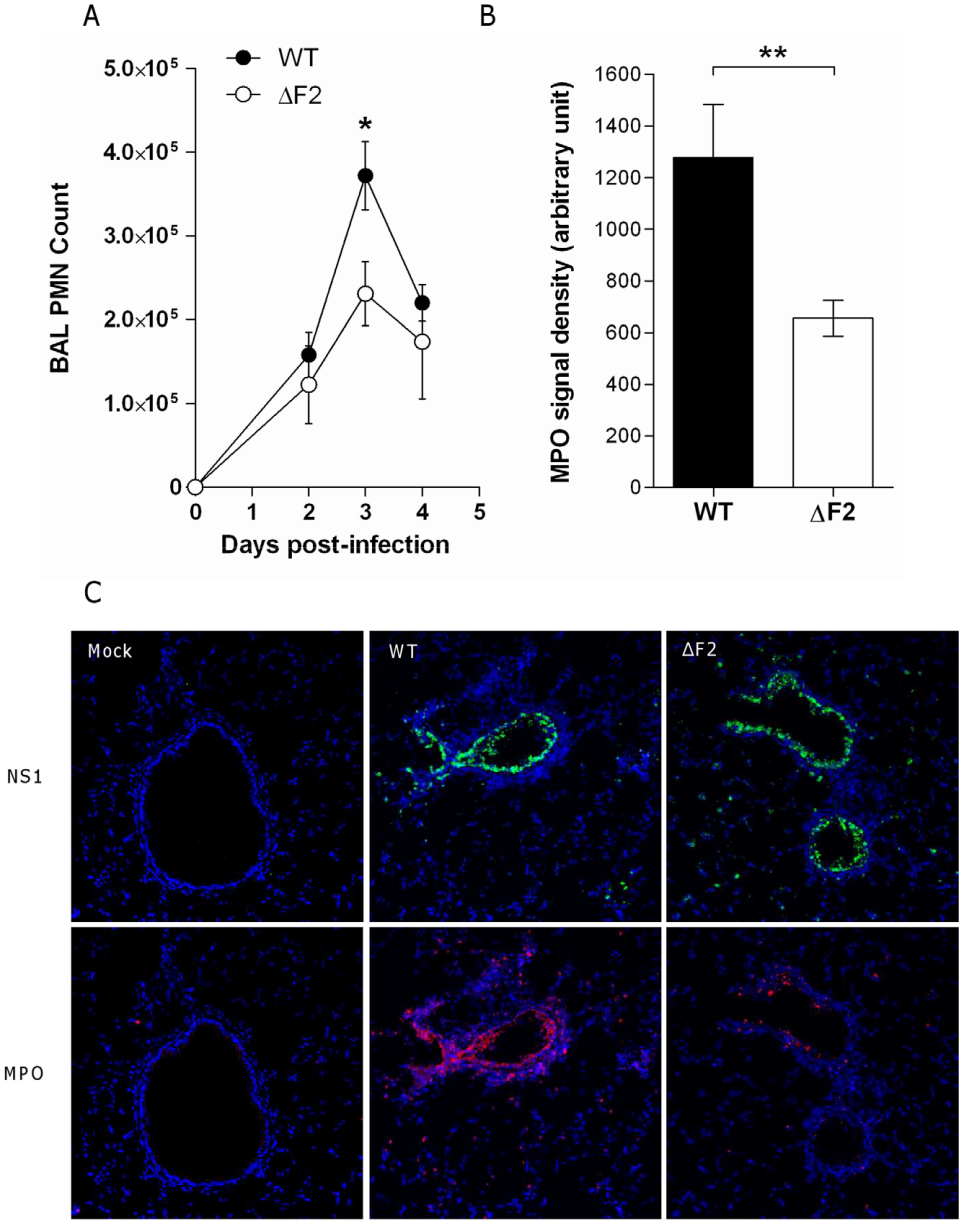
PB1-F2 promotes neutrophil recruitment within IAV-infected mice lungs. (A) Groups of three mice were infected by the wt or the ΔF2 viruses and assayed for number of neutrophils in the BAL fluids after 0, 2, 3 and 4 days pi. (B) In situ immunohistochemistry labeling of the neutrophil marker MPO was assayed on wt-infected and ΔF2-infected 3 days pi. MPO signal density was quantified using “Image J” software. (C) Representative sections of mock-infected (left panels), wt-infected (middle panels) and ΔF2-infected (right panels) mice lungs labeled for NS1 and MPO.

To determine whether PB1-F2 expression could be responsible for an increased recruitment of granulocytes during IAV infection, as suggested by the microarray data, we assessed cellular infiltration in the air spaces. Leukocytes were harvested from the BAL of mock-, wt- and ΔF2-infected mice 3 days pi, the number and phenotype of the cells were characterized by cytospin slide counting. Analysis of the BAL leukocyte populations demonstrated that IAV infections induced the accumulation of polynuclear neutrophils (PNN) in the airways. PNN constitute more than 80 % of the cells found in the BAL fluid of infected mice. The PNN accumulation peaked at day 3 pi and decreased thereafter (Fig.7A). When compared to ΔF2-infected mice, wt-infected mice PNN counts revealed a significantly more important afflux of PNN at day 3 pi (Fig.7A). To further investigate the PB1-F2 effect in the lung tissue, we quantified the myeloperoxidase (MPO, a lysosomal protein specific for PNN) signal at day 3 pi by immunohistology. Infected lung sections presented characteristic histological aspects related to acute inflammation. Staining of the viral NS1 protein showed a characteristic bronchial localization that was homogenous and equivalent in intensity for the two viruses (Fig.7C). Immunohistochemical localization of MPO showed an intense signal in bronchial tissue when mice were infected with the wt virus at day 3 pi. However, when ΔF2-infected mice were analyzed, a weaker MPO signal was observed in the bronchial tissue (Fig.7B, 7C) highlighting the potent pro-inflammatory effect of PB1-F2 during IAV infection. Collectively, these histological analyses designate PB1-F2 as a virulence factor playing a harmful role in the pathogenesis of IAV infection.

## Discussion

Innate immune response is considered as an essential process required for efficient pathogen clearance. However, deregulation of this response can lead to severe damage for the host. The regulation of host defenses to IAV infections is particularly complex and leukocyte recruitment to the inflammatory lung, which is beneficial for clearing the virus, can become deleterious to the host tissues if the reaction is excessive [16]. In fact, although inflammation is essential for IAV clearance, it is becoming increasingly evident that a tight regulation of this process is necessary to avoid development of IAV-related immunopathological sequelae [8], [17]. It illustrates the ambiguity of the host response to infections. The strength of the response has to be proportionate to the pathogen attack in order to eliminate it. If the response is too weak, the pathogen will proliferate, leading to infection. If the host response is too strong, uncontrolled inflammatory reaction will lead to tissue destruction.

The present study demonstrates that PB1-F2 is endowed with pro-inflammatory activity and plays a role in the immunopathological consequences observed in severe impairment of influenza-infected lung function. By the use of reverse genetics, we obtained a mutant virus unable to express PB1-F2 and showed that this mutant was less lethal and virulent than the wt virus, consistent with a previous study [9]. These differences cannot be explained by reduced replication efficiency of the ΔF2 virus since no replication variation was observed between the two viruses. Besides, the use of mutants deleted for N40 or PB1-F2 exclude a potential role of N40 in this process.

Our previous study identified IFN-β gene as a PB1-F2-mediated exacerbated gene during IAV infection of a human respiratory epithelial cell line [6]. Based on these previous results, we hypothesized that PB1-F2 expression may stimulate *in vivo* the host immune response, and as a consequence, be a key element that could explain the higher virulence of a virus expressing PB1-F2. IFN-β measurement within infected-mice lungs confirmed our preliminary *in vitro* observation. To further study whether PB1-F2 could cause immunopathology, we compared the global gene expression patterns of wt- and ΔF2-infected mouse lungs. Our data demonstrate that expression of PB1-F2 during IAV infection enhances the host defense responses by strongly increasing the transcriptional profile of genes involved in inflammation, granulocyte migration and apoptotic pathways. No apoptotic pathways were identified when these two viruses were used to infect human pulmonary epithelial cells *in vitro* [6]. This may reflect the cell-specific behavior of PB1-F2 that only induces apoptotic process within immune cells and not in epithelial cells [2]. Thus, the use of the wt/ΔF2 virus couple showed the ability of PB1-F2 to modulate the host response, contributing to virulence *in vivo*. This is supported by a recent work by McAuley *et al.* characterizing the immunomodulatory properties of several variants of the PB1-F2 protein [5].

Using IPA software, we were able to analyze the functional relationships between the genes differentially regulated when PB1-F2 was expressed or not during infection. The analysis revealed the central role of IFN-γ, which could act as a relay between PB1-F2 and the genes implicated in respiratory disease, inflammatory response, immune cell trafficking, tissue remodeling and apoptosis induction. The PB1-F2-specific increase of IFN-γ expression was confirmed at the protein level in BAL fluids of infected mice. IFN-γ is a member of the type II interferon family and is a potent activator of macrophages and neutrophils. It is worth noting that in the IAV-infected pulmonary environment, IFN-γ represses the innate immunity developed by alveolar macrophages to enhance their adaptive antiviral response through increased MHC gene expression [18]. Consequently, the scavenger receptor MARCO is down-regulated, and innate defense against secondary bacterial infections is suppressed. The link that we identified between PB1-F2 and IFN-γ over-expression appears of first importance since PB1-F2 has been previously described as facilitating the development of opportunistic pneumococcal and staphylococcal infections [12], [19]. Collectively, our data provide a rationale to understand how PB1-F2 exacerbate secondary bacterial infections and post-influenza pneumonia: PB1-F2 induces an apoptosis increase of recruited leucocytes together with a switch of their activation state from innate immunity to adaptive antiviral response through an exacerbation of the IFN-γ expression in the lung.

The mechanism by which PB1-F2 mediates inflammatory increase by itself is still unknown. However, NF-κB pathway exacerbation is involved in this process as shown by the dramatic decrease of NF-κB activity in lungs of mice infected by the ΔF2 virus when compared to the wt infection (Fig. 6). The targeting of PB1-F2 to mitochondria could explain the NF-κB pathway exacerbation through a membrane destabilization of this organelle and activation of the RIG-I/MAVS signaling pathway. However PB1-F2 mitochondrial localization is a matter of debate since several studies described differential localization depending on the strain of the virus and the sequence polymorphism of the protein [20], [21]. The capability of PB1-F2 to perforate or target other cellular membranes could also explain its inflammatory and apoptotic activities [14], [22], [23]. Considering the membrane affinity of PB1-F2, it could form protein pores in endoplasmic reticulum and subsequently induce the release of Ca^2+^ within the cytosol. Such increase in concentration of Ca^2+^ in the cytosol is described to activate the NF-κB pathway and to play a pivotal role in inducing proinflammatory gene transcription in airway epithelial cells [24]. Another important characteristic of PB1-F2 is its propensity to form or promote the formation of amyloïdic fibers in infected monocytes [14]. These fibrillar aggregates are believed to associate to the membrane, disrupt its integrity and lead to perturbation of the cellular compartmentalization. This suggests that PB1-F2 could contribute to the pulmonary dysfunctions observed throughout IAV infections by several pathways.

A striking feature of the inflammation exacerbated by PB1-F2 expression is the enhanced neutrophil recruitment within infected airways. There is evidence, based on their function, that neutrophils play an important role in mediating acute injury characteristic of highly pathogenic IAV infections. Circulating neutrophils migrate to the site of infection and participate in the destruction of pathogens. However this process has to be tightly regulated since the beneficial effect for eliminating microbes can become deleterious to host tissues through the development of lesions. Indeed neutrophils have the potential to damage airspaces by releasing serine proteases and by generating reactive oxygen species [25]. Neutrophil recruitment within lung tissue also increases protein permeability across the endothelial and epithelial barriers of the lung. This leads to the flooding of alveoli by plasma liquid and proteins and is characteristic of early lung injury [26]. However, depletion of neutrophils prior to influenza infection increased viral load and mortality compared to non-treated mice [27], confirming the importance of this type of leucocyte during IAV infection, and underlying the complexity of the host-IAV interactions. A fine balance between inflammation and immunity is necessary to eliminate IAV. Our study shows that PB1-F2 is implicated in the dysfunction of this balance and that it induces a massive recruitment of neutrophils within airspaces through deregulation of the innate host defense.

In summary, the present study demonstrated that PB1-F2 expression significantly increases the expression of genes associated with inflammation in the airways of IAV-infected mice. We identified IFN-γ as a pivotal host component implicated in this process, orchestrating immune cell apoptosis induction, granulocyte recruitment and tissue remodeling observed in infected lungs. The PB1-F2 specific NF-κB pathway exacerbation that we revealed is probably involved in this IFN-γ expression increase. Further studies are required to correlate PB1-F2 sequence variability and IAV virulence to help in the prediction of the pathogenicity of emerging virus strains in the human population.

## Materials and Methods

### Ethics statement

This study was carried out in accordance with INRA guidelines in compliance with European animal welfare regulation. The protocol was approved by the Animal Care and Use Committee at CRJ under relevant institutional (DSV, permit number: 7827) and INRA “Santé Animale” department guidelines. All experimental procedures were performed in a Biosafety level 2 facility.

### Generation of recombinant influenza virus

Influenza A/WSN/1933 (H1N1) was used in this study. Wild type and PB1-F2 knockout viruses were produced using the 12 plasmid reverse genetics system [28]. The viruses were prepared as previously described [14].

### Mice strains

Female C57Bl/6 and Balb/c mice were purchased from the Centre d'Elevage R. Janvier (Le Genest Saint-Isle, France) and were used around 8 weeks of age. Mice strains were bred in an animal facility in pathogen-free conditions. Mice were fed normal mouse chow and water *ad libitum* and were reared and housed under standard conditions with air filtration. For infection experiments, mice were housed in cages inside stainless steel isolation cabinets that were ventilated under negative pressure with HEPA-filtered air.

### Animal infection and fluid collection

Mice were anesthetized by a mixture of ketamine and xylazine (1 and 0.2 mg per mouse, respectively) and infected intranasally with 50 µl of PBS containing 1×10^6^, 5×10^5^, 2.5×10^5^, 1.5×10^5^ or 5×10^4^ plaque forming units (PFU) of IAV. To determine the lethal dose 50% (LD50), groups of 5 mice were infected with different dilutions of virus and observed for signs of morbidity and death over 18 days. Alternatively, mice were killed at different time points, BAL fluids and lungs were then collected as previously described [29].

### RNA extraction and quantitative RT-PCR analysis

Total RNA isolated from mice lungs using Qiagen Rneasy kit (Qiagen) was treated with DNase I and reverse-transcribed with superscript reverse transcriptase (Invitrogen) using random hexamers (Fermentas) or the specific IAV M1 primer : 5′-TCT AAC CGA GGT CGA AAC GTA-3′ for virus quantification [29], [30]. PCR was performed in 20 µl reactions with specific detection primer pairs for mouse IFN-β (sense : 5′-CCC TAT GGA GAT GAC GGA GA-3′ ; antisense : 5′-CTG TCT GCT GGT GGA GTT CA-3′) and IAV M1 (sense : 5′-AAG ACC AAT CCT GTC ACC TCT GA-3′ ; antisense : 5′-CAA AGC GTC TAC GCT GCA GTC C-3′). The mRNA levels of IFN-β and vRNA levels of M1 were assayed using the Mastercycler realplex sequence detector (Eppendorf) and the double strand specific dye SYBR Green system (Applied Biosystems). The PCR conditions and cycles were as follows: initial DNA denaturation 10 min at 95°C, followed by 40 cycles at 95°C for 15 sec, followed by an annealing step at 64°C for 15 sec, and then extension at 72°C during 30 sec. Each point was performed in triplicate. To ensure that the primers produced a single and specific PCR amplification product, a dissociation curve was performed at the end of the PCR cycle. Relative quantitative evaluation was performed by the comparative ΔΔCt method. The mean ΔCt obtained in non stimulated cells for each gene was used as calibrator, after normalization to endogenous control β-actin (sense : 5′-TGT TAC CAA CTG GGA CGA CA-3′ ; antisense : 5′-GGG GTG TTG AAG GTC TCA AA-3′). The results are presented as an n-fold difference relative to calibrator (RQ = 2−ΔΔCt).

### Microarray experiments

Separate microarrays were run for each experimental sample (one sample per mouse and three mice per time point). Transcriptional profiling was performed using Agilent's Whole Mouse Genome Microarray Kit, 4×44 K (G4122F). Experiments were performed at the “Plateau d'Instrumentation et de Compétences en Transcriptomique” (PiCT), INRA Jouy-en-Josas research center. Minimum Information about Microarray Experiment (MIAME) was deposited in ArrayExpress at EMBL (http://www.ebi.ac.uk/microarray-as/ae). A dual color, balanced design was used to provide two direct comparisons: [uninfected/infected-by-wt-virus] and [uninfected/infected-by-ΔF2-virus]. Arrays were hybridized according to the manufacturer's instructions and as previously described [14]. For functional analysis the data files resulting from differential analysis were imported into GeneSpring GX 11 software (Agilent Technologies, Massy, France). Hierarchical clustering analysis was performed to analyze cellular genes that were differentially expressed during infection (Euclidian distance, average linkage). For further analysis, data files were uploaded into the Ingenuity Pathways Analysis (IPA) software (Ingenuity Systems, Redwood City, CA; www.ingenuity.com). Right-tailed Fisher's exact test was used to calculate a p-value determining the probability that each biological function and disease assigned to that data set is due to chance alone.

### ELISA

IL-6, TNF-α, IFN-γ and CXCL1/KC concentrations in mice BAL were determined using DuoSet ELISA kits obtained from R&D Systems (Minneapolis, Minnesota, United States).

### Apoptosis quantification

Apoptosis quantification in cells present in the BAL fluids was performed using the ApoGlow Assay Kit (Lonza) according to the manufacturer's procedures. The assay is based on the bioluminescent measurement of Adenylate Nucleotide Ratio (ADP/ATP).

### *In vivo* Luminescence measurements

Recombinant replication-deficient adenovirus (purchased from “Gene Transfer Vector Core”, University of Iowa) was used to transduce NF-κB-luciferase (Ad-NF-κB-luc) construct in the lung of mice. Ad-NF-κB-luc was prepared as previously described [15]. Mice received intranasal instillations of Ad-NF-κB-luc (2.5×10^8^ PFU). Photon emission of the luminescent construct transducted in the lungs of mock-infected, wt- and ΔF2-IAV-infected mice (1.5×10^5^ PFU per mouse) was measured using the IVIS system (Xenogen Biosciences). A digital false-color photon emission image of the mouse was generated, and photons were counted within a constant defined area corresponding to the surface of the chest encompassing the whole lung region. Photon emission was measured as photons emitted per second.

### Immunolocalization

Paraformaldehyde-fixed cryosections were permeabilized by 0.1% Triton X-100. Endogenous peroxidase activity was inhibited with 3% H_2_O_2_ and nonspecific sites were blocked for 1 hr with 2% bovine serum albumin. Sections were incubated with the relevant primary antibody (goat IgG anti-mMPO R&D Systems, goat anti-NS1, santa cruz biotechnology) for two hours at room temperature and with fluorochrome-conjugated secondary antibodies (bovine anti-IgG goat Cy5, rabbit anti-IgG goat Cy3, Jakson Immuno Research Laboratories) for 1 hr at room temperature. Nuclei were counterstained with DAPI. Double stainings were performed by mixing the primary antibodies and mixing fluorochrome-conjugated reagents, respectively. Quantification of fluorescence staining was made on 6 slides from 3 different mice in each group using Image J software.

### Statistical analysis

Cytokine levels, viral loads, apoptosis measurements, luminescence measurements and PMN counts are expressed as the mean ± standard deviation (SD) of at least three separate replicates, and statistical analyzes were performed using the unpaired Student t-test.

### Accession numbers

Entrez accession number (http://www.ncbi.nlm.nih.gov/Entrez) of the Influenza A virus (A/WSN/1933(H1N1)) segment 2 complete sequence containing PB1-F2 ORF: CY034138. Swiss Prot accession number (http://expasy.org/sprot) of the Influenza A virus (A/WSN/1933(H1N1)) protein PB1-F2 : B4URF6; and PB1 : B4URF5.

## Supporting Information

Figure S1 In vivo kinetics of PB1-F2 expression in IAV-infected mice lungs. Expressions of PB1-F2 and β-actin were monitored by Western blot analysis (Fig1A). β-actin was used as an internal control to standardize protein levels. Band intensities on blots were quantified using Image J software (http://rsbweb.nih.gov/ij/). The value for PB1-F2 are normalized against that obtained for β-actin. Data are presented as ratio of PB1-F2/β-actin amounts and represent the mean ±SEM values obtained from three distinct mice.
(PDF)

Figure S2 **(A)** Table depicting ORF phenotypic summary of segment 2 WSN mutants (adapted from Wise et al. J Virol. 2009.). We used the 12 plasmids reverse genetics system to generate recombinant viruses containing mutations on the segment 2. ΔF2: the initiation and the three in-frame ATG codons of the ORF were mutated to ACG, Stop12: a stop codon were introduced in position 12 of ORF encoding PB1-F2. M40I and M40L mutations destroy the N40 AUG. M40I alters the PB1-F2 sequence (W9L) and M40L is silent in the F2 ORF.**(B)** Lysates from MLE-15 cells mock-infected and infected during 24 h with the different mutant viruses (MOI = 1) were analyzed by Western blotting using an anti-PB1 antibody. The antibody (vC-19 purchased from Santa Cruz Biotechnology) is directed against the C-terminal part of PB1 and is able to recognize PB1 and N40. **(C)** Supernatants of infected MLE-15 cells were assayed for IFN-β secretion by ELISA.
(PDF)

Figure S3 Infectivity of WT and ΔF2 viruses in lungs. **(A)** Mice were infected by 1.5×105 PFU or mock-infected and were sacrificed at day 2 pi. Lungs were dissected and inflated using cryomatrix fluid. Paraformaldehyde-fixed cryosections were then labeled with an anti-NS1 antibody (Santa Cruz Biotechnology) and nuclei were counterstained with DAPI. **(B)** Quantification of fluorescence staining was made on randomly chosen slides from 3 different mice in each group using Image J software. Infectivity of the viruses was compared using the ratio NS1/DAPI, *i.e.* number of infected cells vs.number of total cells of the sections.
(PDF)

Figure S4 Impact of PB1-F2 expression on several inflammatory markers. Total RNA isolated from lungs of infected mice (day 2 pi) were reverse-transcribed and used to quantify expression of several inflammatory markers by qPCR :chemokine (C-C motif) receptor 1 (Ccr1, Gene ID: 12768), chemokine (C-X-C motif) ligand 1 (Cxcl1, Gene ID:14825), colony stimulating factor 3 (Csf3, Gene ID: 12985), pentraxin 3 (Ptx3, Gene ID: 19288), tumor necrosis factor alpha (Tnf-α, Gene ID: 21926) and triggering receptor expressed on myeloid cells 1 (Trem1, Gene ID:58217). Gene expressions were normalized with the β-actin gene expression level and presented as fold increase relative to mock-treated mice. Data are means ± SD obtained from three mice. Asterisks (*) indicates p<0.05.
(PDF)

Figure S5 Positive control of luminescence emission from BALB/c mice intranasally instillated with 2.5×108 PFU of Ad-NF-κB-luc. Mice were anesthetized by a mixture of ketamine and xylazine (1 and 0.2 mg per mouse, respectively) and infected intranasally with 50 µl of PBS containing 2.5×108 PFU of Ad-NF-κB-luc. 24 hours postinhalation, mice were stimulated with 10 µg of E. coli LPS. 24 hours post-stimulation, bioluminescence was measured by intranasal injection of 50 µl of luciferine (500 µg/ml) and capture of photon emission from the chest using the IVIS system. The scale indicates the average radiance: the sum of the ph otons per second from eachpixel inside the ROI/number of pixels (photons/sec/cm2/sr).
(PDF)
