# Supplementary material for: Transcriptomic Analysis of Host Immune and Cell Death Responses Associated with the Influenza A Virus PB1-F2 Protein
Source: PLoS Pathog. 2011 Aug 25;7(8):e1002202. doi: 10.1371/journal.ppat.1002202 (PMC3161975; doi:10.1371/journal.ppat.1002202)
Supplement: Figure S1 — In vivo kinetics of PB1-F2 expression in IAV-infected mice lungs. Expressions of PB1-F2 and β-actin were monitored by Western blot analysis (Fig1A). β-actin was used as an internal control to standardize protein levels. Band intensities on blots were quantified using Image J software (http://rsbweb.nih.gov/ij/). The value for PB1-F2 are normalized against that obtained for β-actin. Data are presented as ratio of PB1-F2/β-actin amounts and represent the mean ±SEM values obtained from three distinct mice. (PDF) [file ppat.1002202.s001.pdf]

Figure S1. Le Goffic *et al.*

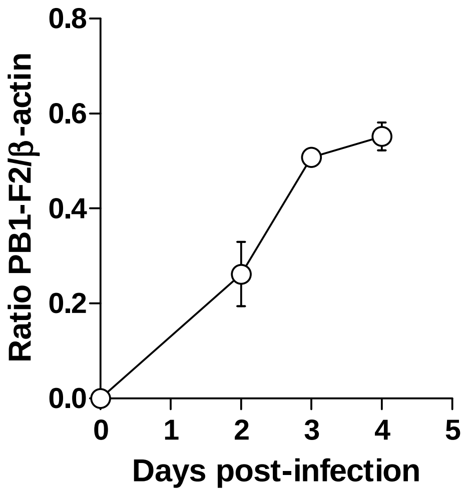

Supplemental Figure S1:

In vivo kinetics of PB1-F2 expression in IAV-infected mice lungs. Expressions of PB1-F2 and β-actin were monitored by Western blot analysis (Fig1A). β-actin was used as an internal control to standardize protein levels. Band intensities on blots were quantified using Image J software (http://rsbweb.nih.gov/ij/). The value for PB1-F2 are normalized against that obtained for β-actin. Data are presented as ratio of PB1-F2/β-actin amounts and represent the mean ±SEM values obtained from three distinct mice.
