# Supplementary material for: Transcriptomic Analysis of Host Immune and Cell Death Responses Associated with the Influenza A Virus PB1-F2 Protein
Source: PLoS Pathog. 2011 Aug 25;7(8):e1002202. doi: 10.1371/journal.ppat.1002202 (PMC3161975; doi:10.1371/journal.ppat.1002202)
Supplement: Figure S2 — (A) Table depicting ORF phenotypic summary of segment 2 WSN mutants (adapted from Wise et al. J Virol. 2009.). We used the 12 plasmids reverse genetics system to generate recombinant viruses containing mutations on the segment 2. ΔF2: the initiation and the three in-frame ATG codons of the ORF were mutated to ACG, Stop12: a stop codon were introduced in position 12 of ORF encoding PB1-F2. M40I and M40L mutations destroy the N40 AUG. M40I alters the PB1-F2 sequence (W9L) and M40L is silent in the F2 ORF.(B) Lysates from MLE-15 cells mock-infected and infected during 24 h with the different mutant viruses (MOI = 1) were analyzed by Western blotting using an anti-PB1 antibody. The antibody (vC-19 purchased from Santa Cruz Biotechnology) is directed against the C-terminal part of PB1 and is able to recognize PB1 and N40. (C) Supernatants of infected MLE-15 cells were assayed for IFN-β secretion by ELISA. (PDF) [file ppat.1002202.s002.pdf]

Figure S2. Le Goffic *et al.*

A

	Effect on ORF		
Virus	PB1	PB1-F2	N40
WSNΔF2	WT	Null	WT
WSN-F2stop12	WT	11 aa	WT
WSN-M40I	M40I	W9L	Null
WSN-M40L	M40L	WT	Null

B

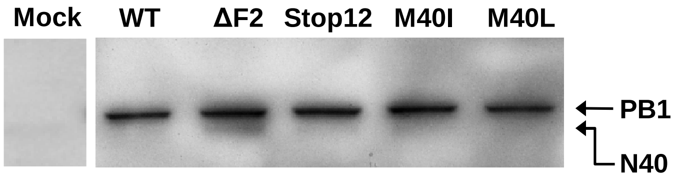

C

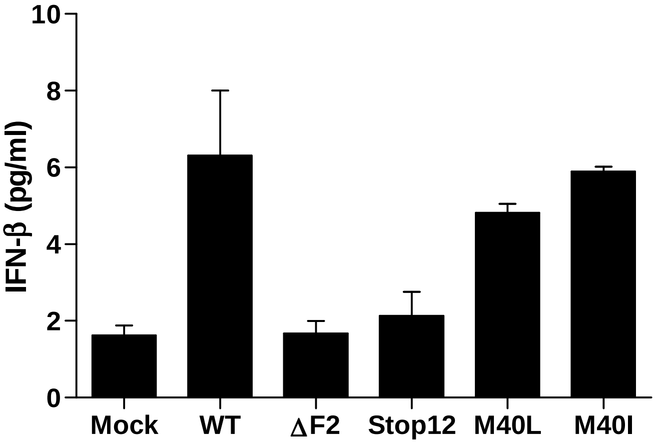

Supplemental Figure S2:
(A) Table depicting ORF phenotypic summary of segment 2 WSN mutants (adapted from Wise et *al.* J Virol. 2009.). We used the 12 plasmids reverse genetics system to generate recombinant viruses containing mutations on the segment 2. ΔF2 : the initiation and the three in-frame ATG codons of the ORF were mutated to ACG, Stop12 : a stop codon were introduced in position 12 of ORF encoding PB1-F2. M40I and M40L mutations destroy the N40 AUG. M40I alters the PB1-F2 sequence (W9L) and M40L is silent in the F2 ORF.(B) Lysates from MLE-15 cells mock-infected and infected during 24h with the different mutant viruses (MOI=1) were analyzed by Western blotting using an anti-PB1 antibody. The antibody (vC-19 purchased from Santa Cruz Biotechnology) is directed against the C-terminal part of PB1 and is able to recognize PB1 and N40. (C) Supernatants of infected MLE-15 cells were assayed for IFN-β secretion by ELISA.
