# Supplementary figures and images for: Transcriptomic Analysis of Host Immune and Cell Death Responses Associated with the Influenza A Virus PB1-F2 Protein
Source: PLoS Pathog. 2011 Aug 25;7(8):e1002202. doi: 10.1371/journal.ppat.1002202 (PMC3161975; doi:10.1371/journal.ppat.1002202)

Figure S3. Le Goffic *et al.*

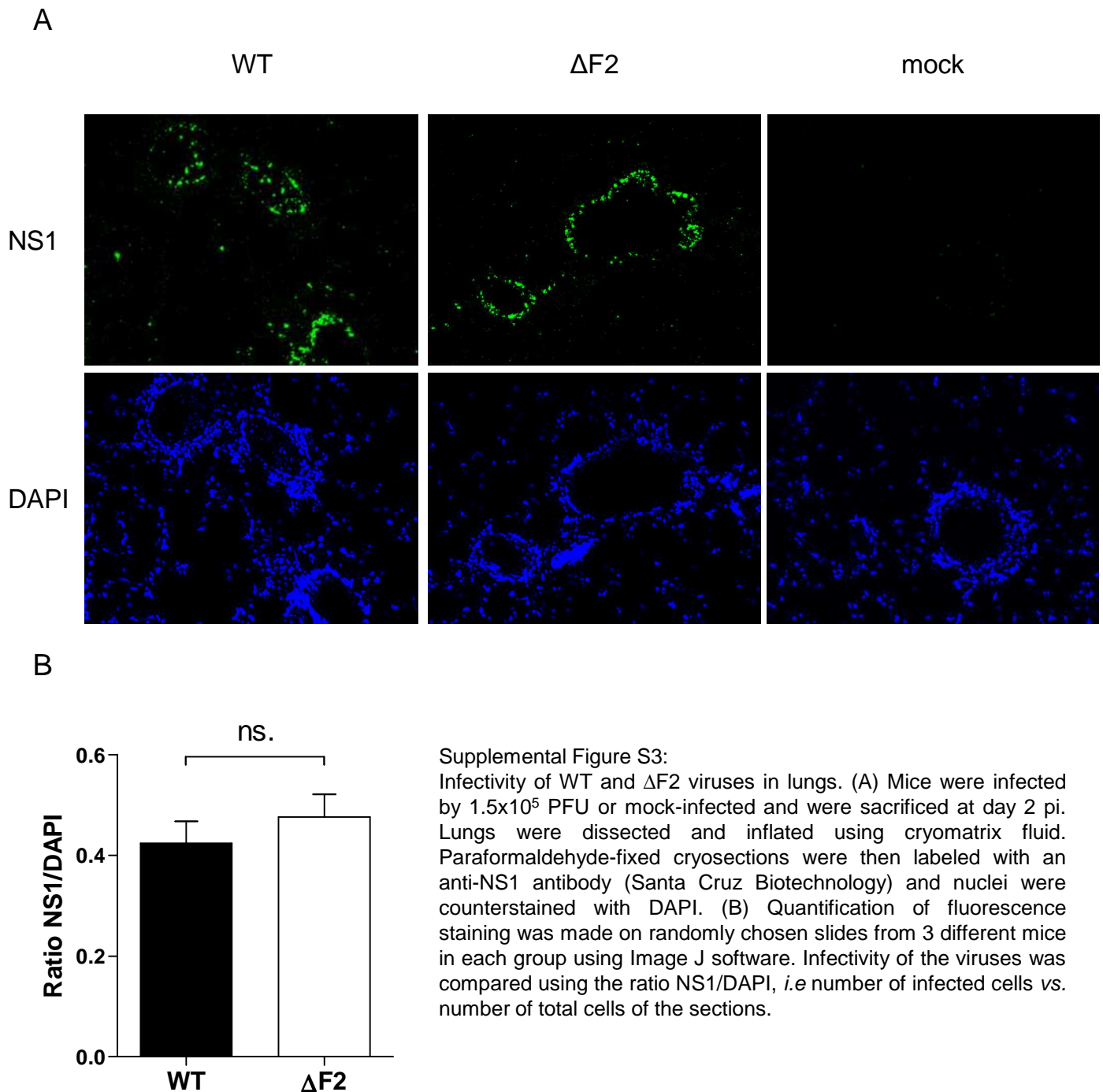

Supplement: Figure S3 — Infectivity of WT and ΔF2 viruses in lungs. (A) Mice were infected by 1.5×10^5^ PFU or mock-infected and were sacrificed at day 2 pi. Lungs were dissected and inflated using cryomatrix fluid. Paraformaldehyde-fixed cryosections were then labeled with an anti-NS1 antibody (Santa Cruz Biotechnology) and nuclei were counterstained with DAPI. (B) Quantification of fluorescence staining was made on randomly chosen slides from 3 different mice in each group using Image J software. Infectivity of the viruses was compared using the ratio NS1/DAPI, i.e. number of infected cells vs.number of total cells of the sections. (PDF) [file ppat.1002202.s003.pdf]
