# Supplementary material for: Transcriptomic Analysis of Host Immune and Cell Death Responses Associated with the Influenza A Virus PB1-F2 Protein
Source: PLoS Pathog. 2011 Aug 25;7(8):e1002202. doi: 10.1371/journal.ppat.1002202 (PMC3161975; doi:10.1371/journal.ppat.1002202)
Supplement: Figure S4 — Impact of PB1-F2 expression on several inflammatory markers. Total RNA isolated from lungs of infected mice (day 2 pi) were reverse-transcribed and used to quantify expression of several inflammatory markers by qPCR :chemokine (C-C motif) receptor 1 (Ccr1, Gene ID: 12768), chemokine (C-X-C motif) ligand 1 (Cxcl1, Gene ID:14825), colony stimulating factor 3 (Csf3, Gene ID: 12985), pentraxin 3 (Ptx3, Gene ID: 19288), tumor necrosis factor alpha (Tnf-α, Gene ID: 21926) and triggering receptor expressed on myeloid cells 1 (Trem1, Gene ID:58217). Gene expressions were normalized with the β-actin gene expression level and presented as fold increase relative to mock-treated mice. Data are means ± SD obtained from three mice. Asterisks (*) indicates p<0.05. (PDF) [file ppat.1002202.s004.pdf]

Figure S4. Le Goffic *et al.*

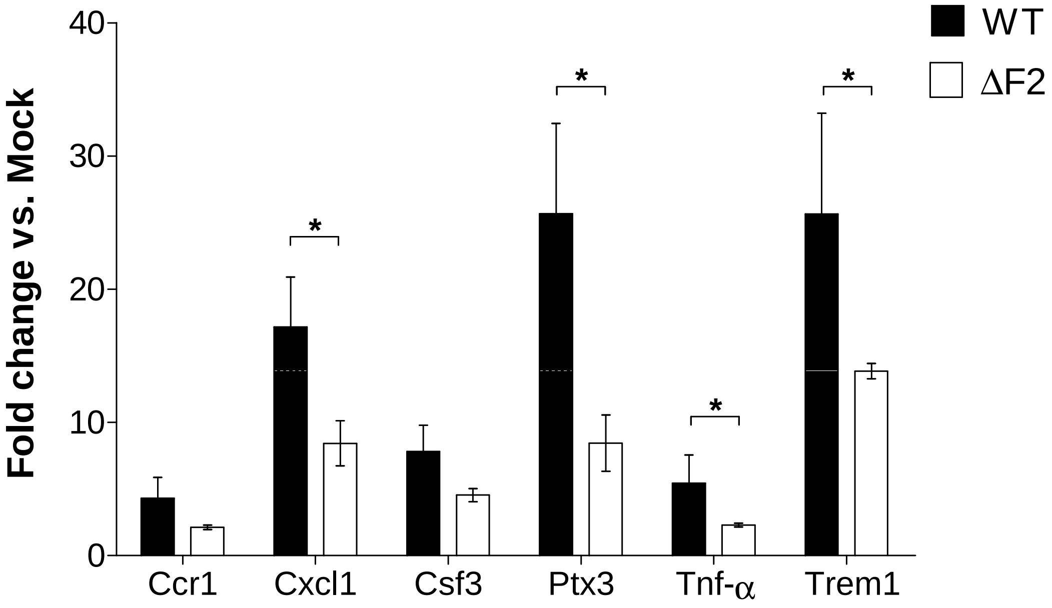

Supplemental Figure S4:

Impact of PB1-F2 expression on several inflammatory markers. Total RNA isolated from lungs of infected mice (day 2pi) were reverse-transcribed and used to quantify expression of several inflammatory markers by qPCR : chemokine (C-C motif) receptor 1 (Ccr1, Gene ID: 12768), chemokine (C-X-C motif) ligand 1 (Cxcl1, Gene ID: 14825), colony stimulating factor 3 (Csf3, Gene ID: 12985), pentraxin 3 (Ptx3, Gene ID: 19288), tumor necrosis factor alpha (Tnf-α, Gene ID: 21926) and triggering receptor expressed on myeloid cells 1 (Trem1, Gene ID: 58217). Gene expressions were normalized with the β-actin gene expression level and presented as fold increase relative to mock-treated mice. Data are means ± SD obtained from three mice. Asterisks (*) indicates $p<0.05$.
