# Supplementary material for: Transcriptomic Analysis of Host Immune and Cell Death Responses Associated with the Influenza A Virus PB1-F2 Protein
Source: PLoS Pathog. 2011 Aug 25;7(8):e1002202. doi: 10.1371/journal.ppat.1002202 (PMC3161975; doi:10.1371/journal.ppat.1002202)
Supplement: Figure S5 — Positive control of luminescence emission from BALB/c mice intranasally instillated with 2.5×10^8^ PFU of Ad-NF-κB-luc. Mice were anesthetized by a mixture of ketamine and xylazine (1 and 0.2 mg per mouse, respectively) and infected intranasally with 50 µl of PBS containing 2.5×10^8^ PFU of Ad-NF-κB-luc. 24 hours postinhalation, mice were stimulated with 10 µg of E. coli LPS. 24 hours post-stimulation, bioluminescence was measured by intranasal injection of 50 µl of luciferine (500 µg/ml) and capture of photon emission from the chest using the IVIS system. The scale indicates the average radiance: the sum of the ph otons per second from eachpixel inside the ROI/number of pixels (photons/sec/cm2/sr). (PDF) [file ppat.1002202.s005.pdf]

Figure S5. Le Goffic *et al.*

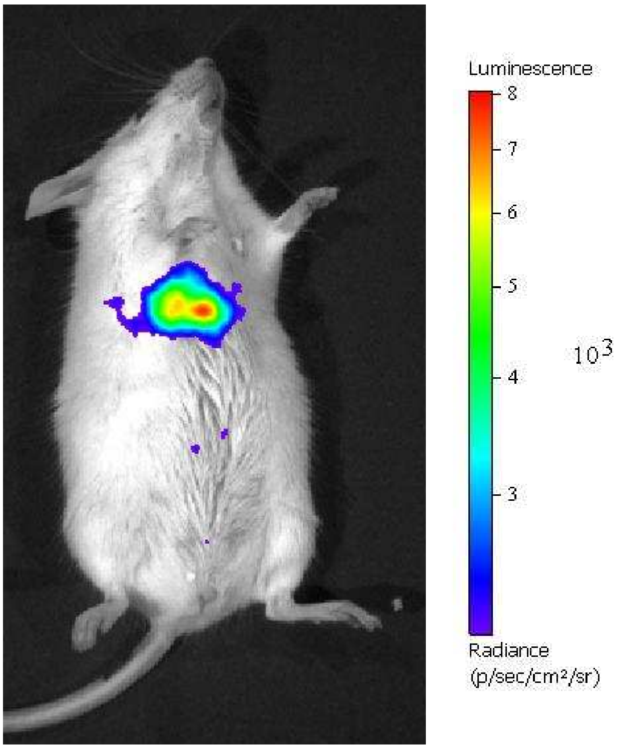

Supplemental Figure S5:
Positive control of luminescence emission from BALB/c mice intranasally instillated with $2.5x10^8$ PFU of Ad-NF-κB-luc. Mice were anesthetized by a mixture of ketamine and xylazine (1 and 0.2 mg per mouse, respectively) and infected intranasally with 50 µl of PBS containing $2.5x10^8$ PFU of Ad-NF-κB-luc. 24 hours post-inhalation, mice were stimulated with 10µg of E. coli LPS. 24 hours post-stimulation, bioluminescence was measured by intranasal injection of 50µl of luciferine (500µg/ml) and capture of photon emission from the chest using the IVIS system. The scale indicates the average radiance: the sum of the photons per second from each pixel inside the ROI/number of pixels (photons/sec/cm2/sr).
